# Social Norms in Cervical Cancer Screening

**DOI:** 10.1177/00332941231219943

**Published:** 2023-12-04

**Authors:** Sarah Wilding, Daryl B. O’Connor, Mark Conner

**Affiliations:** School of Psychology, 4468 University of Leeds, Leeds, UK

**Keywords:** Cervical cancer screening, social norms, behaviour change

## Abstract

Cancer screening aims to check the body for cancer before symptoms develop. Social norms theory suggests people falsely perceive the attitudes and/or behaviours of similar others to be different from their own and correcting these perceptions can lead to behaviour change. Across two studies, we tested if women underestimate peer levels of cervical screening behaviour and whether a social norms manipulation increases intention to attend cervical cancer screening. In study 1, participants completed a survey on cervical cancer screening norms. In study 2, participants were randomised to receive no norm information, norm information, or norm information plus statement on value of norms in decision making. In study 1, participant estimates of peer level of cervical screening behaviour were significantly lower than nationally reported levels. In study 2, a social norm plus value statement intervention led to stronger intentions to attend screening. This effect was consistent across demographic factors and screening status. Participants significantly underestimate rates of cervical screening behaviour in their peers. A brief, online social norms plus values manipulation increased intentions to attend cervical cancer screening across all groups.

## Introduction

Cancer screening aims to check the body for cancer before any symptoms develop. This can help to diagnose and treat cancer at an early stage (Cancer Research UK, 2018) and contribute to reducing overall mortality. The Centres for Disease Control in the USA recommends regular screening for cervical, breast and colorectal cancers (Centres for Disease Control, 2019), although these behaviours can also lead to over-diagnosis, false positives and associated negative effects including over-treatment and anxiety (Marmot et al., 2012; Myers et al., 2015). In England, the NHS offers routine screening for cervical cancer for women aged 25–64 years (NHS, 2018). Women aged 25–49 receive a screening invitation every 3 years and women aged 50–64 receive an invitation every 5 years. Cervical cancer screening is estimated to prevent 70% of cervical cancer deaths, and if everyone attended this could prevent 83% of cervical cancer deaths (Landy et al., 2018). However, current rates of cervical screening in the UK are suboptimal with the published figures suggesting only 70.2% of invited individuals attended screening in 2020-21 (NHS Digital, 2019; Public Health England, 2018). Cervical cancer screening is suggested to be associated with age, socioeconomic status (SES) and ethnicity. Rates are lowest in the youngest age groups of invitees (25–29, Cancer Research UK, 2018), ethnic minority group populations, and those from low socioeconomic status areas (Douglas et al., 2016; NHS Digital, 2019; Wilding et al., 2020, 2022).

Social norms theory suggests that people often falsely perceive (i.e., under- or over-estimate) the attitudes and/or behaviours of important others to be different from their own (Berkowitz, 2005; Schultz et al., 2007). There is a large amount of research investigating social norms theory and interventions using a social norms approach (SNA) in health risk behaviours such as alcohol and drug use (e.g., Burchell et al., 2013; Lewis & Neighbors, 2006; Perkins, 2014). These studies measure estimates of rates of others performance of behaviours and compare these to self-estimates or objective estimates of engagement and show that individuals tend to overestimate performance of these risk behaviours by others. Such studies also show that providing feedback on self-estimates or objective measures of behaviour can lead to reductions in these behaviours among those who overestimate. Studies using models such as the Theory of Planned Behaviour to predict intention and screening behaviour also support the influence of norms (Sieverding, Matterne, & Ciccarello, 2010). One study demonstrated that injunctive norms (whether individuals perceive that others think they should perform the behaviour) were the strongest predictor of the prostate specific antigen (PSA) test (for prostate cancer) and colon cancer screening intentions; injunctive norms were also a significant predictor of intentions to get a mammogram (Smith-McLallen & Fishbein, 2008; Stok et al., 2014).

Fewer studies have investigated the social norms approach in cancer screening behaviours (Sieverding, Decker, & Zimmermann, 2010; Stoffel et al., 2021; Stoffel et al., 2019; von Wagner et al., 2019), particularly cervical cancer screening. It has been proposed that individuals tend to underestimate the extent to which their peers engage in such health promotion/protection behaviours (Perkins, 2014), although estimated levels of behaviour are associated with own behaviour whereby individuals who estimate rates to be higher tend to be those who regularly engage with the behaviour themselves (Sieverding, Matterne, & Ciccarello, 2010). Underestimation of the rates of health promotion behaviours may be an important factor in discouraging performance of these behaviours. SNA interventions rely on providing information regarding the behaviour or cognitions relating to a specific behaviour in people similar to the individual. Interventions are more successful when the information provided relates to peers who individuals can identify with in terms of specific characteristics such as being of a similar age or gender (Dempsey et al., 2018).

As outlined earlier, cervical cancer screening is suggested to be associated with age, socioeconomic status (SES) and ethnicity. For example, UK national data has shown that cervical screening uptake is lowest in the youngest groups along with the oldest age groups of invitees (Cancer Research UK, 2018; NHS Digital, 2019). It has been suggested that these differential rates may exist due to different barriers, with younger women reporting practical barriers such as a lack of time or issues with childcare, while older women report attitudinal barriers to attendance (e.g., low worry or perceived risk to cervical cancer; Waller et al., 2012). Uptake is also suggested to be associated with ethnicity. While national data does not record uptake statistics by ethnicity, it has been consistently reported that ethnic minority groups report additional barriers to screening (Douglas et al., 2016; NHS Digital, 2019). One study found that minority ethnic women were more than twice as likely to have never attended screening in the UK compared to white women (Moser et al., 2009). In terms of socioeconomic status, rates of uptake have been found to be consistently lower in individuals from the most deprived backgrounds compared to those from the least deprived backgrounds (NHS Digital, 2019; Douglas et al., 2016; NHS). Therefore, given these reported differences in uptake by age, ethnicity and socioeconomic status, the current studies aimed to explore the potential role of these factors in this research.

The present research had two main aims. First, to assess the extent to which UK women underestimate levels of cervical cancer screening behaviour in their peers and the extent to which other factors (i.e., women’s own age group, socio-economic status group, ethnicity, screening past behaviour) moderate such estimates. Second, to assess whether providing accurate information about peers’ levels of cervical cancer screening behaviour changes intentions to get screened and the extent to which other factors (i.e., specific peer group rated; women’s own age group, socio-economic status group, ethnicity, current screening status) moderate any effects. Study 1 addressed the first of these aims, while Study 2 addressed the second of these aims.

## Study 1

Study 1 aimed to investigate differences between estimated cervical cancer screening rates in a sample of women eligible for cervical cancer screening in the UK. It also aimed to assess whether women’s own socio-demographic characteristics (age group, SES group, ethnicity) or screening status affected these judgements.

### Materials and Method

#### Design and Participants

1074 participants were considered for inclusion in the study based on age, ethnicity and SES. We aimed to recruit 500 participants in total to the final survey with roughly equal numbers of younger (aged 25–49) and older (aged 50–64) participants split into equal numbers of high versus low levels of SES. In addition, we aimed to ensure that 20% of the recruited sample were from minority ethnic groups, in order to match the breakdown of ethnic groups in the UK population (UK Government, 2011), again the participants from each ethnic grouping were stratified by age and SES.

A total of 500 respondents completed the final survey; all were women living in the United Kingdom. They were recruited in October 2021 via Prolific (https://www.prolific.com/), an online research recruitment website. All participants were living in the UK, and each of the four UK nations was represented. Based on self-reported postcode, an Index of Multiple Deprivation (IMD) decile was calculated using the postcode lookup provided by each of the UK nations (Government, 2021; Ministry of Housing, 2021; Northern Ireland Statistics & Research Agency, 2021; Scottish Government, 2021). A total of 254 (50.8%) respondents were coded into the most deprived five deciles and 246 (49.2%) were coded into the least deprived five deciles. 52.6% of participants (*N* = 263/500) were educated to undergraduate degree level or higher. Of the 498 participants reporting valid ethnicity, 397 participants (79.4%) were white, and 101 participants (20.2%) were from minority ethnic groups. The study received ethical approval from the University Department’s Ethics Committee.

A total of 407 participants (81.4%) reported they were currently up to date with their cervical cancer screening (i.e., had attended in the past 3 years if aged 25–49 or in the past 5 years if aged 50–64).

#### Measures

Participants completed measures to tap demographic information including education, age, ethnicity and postcode (which was converted into IMD). They also reported when they last attended for cervical screening (past behaviour), this was dichotomised using this item. This was coded based on age where participants aged 25–49 years who reported they had been screened in the past 3 years were classified as up-to-date with screening, as were participants aged 50–64 who reported they had been screened in the past 5 years. All other responses were classified as overdue/never screened.

Participants were asked to estimate the proportion of individuals ‘like you’ who had ever participated in cervical screening behaviour rated between 0%–100% (e.g., “What percentage of women like you do you think have ever in their lifetime been for cervical cancer screening?”). Participants were then asked to report the proportion of individuals ‘like you’ who had participated in cervical cancer screening in the past 3–5 years (e.g., “What percentage of women like you do you think have been for cervical cancer screening in the past 3–5 years?”)

Screening intention was also assessed using 3 items: “Do you intend to go for cervical cancer screening when you are next invited?”; “Do you plan to go for cervical cancer screening when you are next invited?” Definitely don’t-Definitely do; “Will you go for cervical cancer screening when you are next invited?” Definitely won’t- Definitely will; α = .995). These were rated on seven-point Likert scales and coded so that high scores reflected high intention. Ratings were then standardised and averaged to create a mean intention score.

A number of other measures were also taken but are not reported here but form part of another publication. Full copies of the questionnaire can be obtained from the first author.

#### Procedure

Respondents were recruited via Prolific (https://prolific.ac/) and after screening they were invited to take part in a ‘cancer screening survey’. They gave informed consent and were then asked to complete the questionnaire via Qualtrics. On completion they were thanked and paid £1.25 for completing a 15-min survey.

#### Analyses

Mean estimated rates of cervical cancer screening were compared against national rates of screening for that group using one-sample t-tests. MANOVA was then used to investigate differences in perceived screening rates by age group (aged 25–49; 50–64), ethnicity (white; minority ethnic group), IMD group (most deprived 5 deciles; least deprived 5 deciles) and recency of screening (up-to-date; overdue/never screened) plus the interactions. Regression analyses were then used to assess the relationship between the difference between the two estimations and the average screening rate (group adjusted by age) as predictors of screening intention. Note that these regression analyses were performed separately for the two outcomes (i.e., one analysis for the difference between the national average and the estimations of ever been screened and a second analysis for the difference between the national average and estimations of having been screened in the last 3–5 years).

### Results

#### Perceived Cervical Screening Behaviour

Women tended to significantly underestimate the rates of cervical cancer screening uptake of other women compared to the national averages. Compared to a national average figure across all eligible women of 70.2% up to date with screening, it was estimated that 68.3% (SD = 16.8) of women had ever attended screening, *t*(499) = −2.71, *p* = .007; *d* = −.12, 95% CI [−.21, −.03]. Estimates of the proportion of women up to date with screening (had attended in the past 3–5 years) was even lower (60.5%; SD = 18.6) and was just under 10% lower than the actual figure, indicating, as per Cohen’s (1988) criteria, a moderate sized underestimation effect regarding the proportion of women that attend screening, *t*(499) = −11.71, *p* < .001 *d* = −.52, 95% CI [−.62, −.43].

MANOVA indicated that estimates of women’s cervical screening rates did differ by the age group of the participants for both the estimation of whether women had ever been screened (F(1, 481) = 9.32, *p* = .002), and the estimated percentage of women that were up-to-date with screening (*F*(1, 481) = 7.49, *p* = .006), with younger women reporting lower estimates (M = 64.9, SD = 15.71 and M = 56.8, SD = 18.9) compared to older women (M = 71.75, SD = 15.22 and M = 64.22, SD = 17.57). Moreover, the level that participants underestimated rates in their peers was significantly different from the published screening rates in both age groups except for older women when estimating rates for the ever been screened variable: younger women ever screened: t(249) = −5.31, *p* < .001; *d* = −.34, 95% CI [-.46, −.21]; up-to-date: t(249) = −11.18, *p* < .001; *d* = −.71, 95% CI [-.85, −.57]; older women ever screened t(247) = 1.61, *p* = .055; *d* = .10, 95% CI [-.02, .23]; up-to-date: t(247) = - 5.35, *p* < .001; *d* = −.34, 95% CI [−.47, −.21].

Estimates also significantly differed by IMD group, *F*(1, 481) = 7.65, *p* = .006, and *F*(1, 481) = 6.62, *p* = .010, with individuals from more deprived areas reporting lower estimates (M = 65.9, SD = 16.47; M = 58.64, SD = 18.85) compared to individuals from less deprived areas (M = 70.74, SD = 14.69; M = 62.30, SD = 18.16). The level that participants underestimated rates in their peers was significantly different from the published screening rates in all cases except for women in the least deprived group estimating rates of ever been screened. Women from more deprived areas ever screened: t(253) = −4.15, *p* < .001; *d* = −.26, 95% CI [−.38, −.14]; up-to-date: t(253) = −9.77, *p* < .001; *d* = −.61, 95% CI [−.75, −.48]; Women from least deprived areas ever screened: t(242) = .57, *p* = .28; *d* = .04, 95% CI [−.09, .16]; up-to-date: t(242) = − 6.78, *p* < .001; *d* = −.44, 95% CI [−.57, −.30]. The estimated rates did not differ by ethnicity or by own cervical screening past behaviour (*p* > .16) and none of the interactions between the variables were statistically significant (ps > .14).

The relationship between the difference between the two estimations and the average screening rate (group adjusted by age) as predictors of screening intention was then assessed using regression. Age group, ethnicity and IMD were controlled for in the analysis. Age was a significant predictor of intention (β = −.378, SE = .089, *p* < .001) as was IMD (β = .179, SE = .088, *p* = .04) as was the size of difference between average screening rate and the estimated proportion of women currently up to date with screening (β = −.008, SE = .003, *p* = .009), suggesting a larger difference between the estimated and average rate was associated with lower screening intentions. Ethnicity (*p* = .06) and the estimated proportion of ever screened women (*p* = .74) were not significant predictors of intention. The interaction effects between demographic variables and difference in screening estimates were entered in a final step but none of these interactions were found to be significant (*p* > .17).

### Discussion

Participants significantly underestimated rates of cervical screening behaviour compared to national averages. Regression analyses also supported the idea that a larger difference between estimated and actual percentages of women currently up to date with screening (i.e., greater inaccuracy in estimates) was associated with lower screening intentions. This supports social norm theory (Berkowitz, 2005; Schultz et al., 2007) and the idea that individuals are generally poor at estimating rates of behaviour in others. It also suggests that greater inaccuracy is associated with weaker screening intentions. As expected, the perceived estimated behaviour of others was lower than national data for this key screening behaviour. Estimates also differed depending on the age group and socioeconomic status of individuals, with younger women and those from areas of greater deprivation reporting lower estimates. No differences were found by women’s ethnicity or past behaviour.

We did not find a main effect of screening past behaviour (whether individuals were currently up to date with screening) on the estimated levels that peers engaged in screening behaviour. This does not support previous research suggesting that estimations are associated with individuals’ own behaviour whereby higher estimated levels tend to be provided by people who regularly engage with the behaviour (Sieverding, Matterne, & Ciccarello, 2010).

## Study 2

Study 2 aimed to investigate whether providing feedback on the actual levels of peer behaviour would influence intentions to attend cervical cancer screening when next invited, in a group of women living in the UK who were eligible for cervical cancer screening. Due to the prolonged time interval between screening invitations (3 years in women aged 25–49 and 5 years in women aged 50–64), it was not feasible to assess actual screening behaviour and therefore screening intentions were assessed as the outcome measure. The research also explored whether information indicating the potential value of normative information in making a decision about cervical screening would enhance the effects of providing any normative information. In particular, we used a manipulation of value that was similar to that used by Snyder and Kendzierski (1982) in relation to attitudes. In the Snyder and Kendzierski (1982) study it was shown that informing participants that their attitude was relevant to the decision increased the correspondence between attitudes and behaviour for those both low (i.e., look inwardly in making decisions) and high (i.e., look to their social environment in making decisions) in self-monitoring. Moreover, this study also showed that techniques that increase the relevance of attitudes (by influencing importance and connectedness) “enhance correspondence between attitude and behavior to the extent that they successfully induce individuals to adopt a “believing means doing” orientation to choosing their actions” (Snyder & Kendzierski, 1982; p. 181). This orientation provides individuals with a ‘plan’ to link their attitudes to their behaviours.

Study 2 was designed to test whether providing data on actual rates of cervical screening and the way this was presented could influence intentions to attend cervical cancer screening in future. Considering that a number of studies have demonstrated that providing social norms information alone is not enough to influence behaviour change (e.g. Stoffel et al., 2021; Wilding et al., 2020, 2023), we additionally tested the effect of increasing the salience of the norm messaging, by highlighting the potential impact that providing normative information can have.

Therefore, taken together, it was hypothesised that: (i) the norms only and the norms + value conditions would lead to greater screening intentions compared to the control condition (while controlling for age, recency of screening, IMD quintile, ethnicity); and (ii) the intervention effectiveness would vary by age, ethnicity, IMD quintile and recency of cervical screening attendance.

### Materials and Method

#### Design and Participants

A control condition (no feedback) and two experimental conditions (feedback) were compared. Both experimental groups were presented with age-specific information that was tailored to their age group. In one experimental group (norms + value) the feedback was supplemented with information highlighting that normative information may be of value in deciding whether to engage in cervical screening. Participants were screened prior to taking part and we aimed to recruit 50% participants with (a) low intention to attend screening, or (b) who were overdue for screening/have never attended in the past. The other 50% of participants were those that were currently up-to-date with screening.

In Prolific, 600 female participants aged 25–64 years, currently residing in the UK, were considered for inclusion in the study based on their past screening behaviour and intention to screen in the future. A total of 314 women were deemed eligible for the study based on previous screening history and intention to attend screening when next invited (134 overdue/never screened/low intention women; 184 up-to-date/high intention women). However, only 300 study places were made available via Prolific in order to reduce likelihood of attrition between the screening survey and main study survey. The sample size was selected to have high power (>80%) to detect small sized differences between the different conditions with alpha = .05.

In the main survey, a total of 299 women were randomised to condition, completed the survey and provided valid responses to some measures. 55 (18.41%) of the women had recently (in last year) attended cervical screening. 148 (49.5%) of the women were classified as weak intenders (see below for details of measure). The 299 participants ranged between 25 and 64 years (Mean = 36.5, SD = 10.16). 215 (71.9%) of the women provided a valid postcode and had an IMD quintile calculated (the remaining 84 women were coded as being in the middle quintile for analysis purposes). 83 (27.8%) were coded into the lower two quintiles, and 91 (30.4%) into the higher two quintiles. 295 (98.7%) of the women reported their ethnicity (the remaining 4 women were coded into the largest group – white - for analysis purposes). 258 (86.3) women were coded as white. The study received ethical approval from the University Department’s Ethics Committee.

#### Measures and Materials

In each condition, participants reported how recently they had attended for cervical cancer screening, age, postcode and ethnicity. Reactance was assessed in all conditions using the three-item measure developed by Hall et al., (2017) in order to assess whether there were any differences in participants’ resistance to the health messages by condition, and if so, to consider including it as a potential covariate (“This message is trying to manipulate me”; “The message effect of this warning is overblown”; “This message annoys me”) on a 5-point scale (strongly disagree to strongly agree, α = .67). In addition, intention to attend for cervical screening in the future was also assessed. Five intention items were used. Participants were asked to report their intentions to attend cervical cancer when next invited (“I expect to…”, “I plan to…”, “I will…”, “I want to…” “go for cervical cancer screening when I am next invited”). This was rated on a ten-point scale anchored between strongly disagree to strongly agree. Screening commitment was assessed by participants indicating between 0% and 100% how committed they were to attending screening when next invited. These five items were standardized and averaged (α = .98). However, responses were non-normally distributed and therefore a dichotomous measure of intention was created by splitting at the median (151 low intenders scored 0; 148 high intenders scored 1).

In the control condition no additional information was provided. In the norms condition women were provided with tailored feedback on the rates of cervical screening in their age group (“*In UK women aged… 25-29, 61% are up-to-date with screening, 94% believe cervical cancer screening is important; 30-49, 71% are up-to-date with screening, 89% believe cervical cancer screening is important; 50-64, 74% are up-to-date with screening; 89% believe cervical cancer screening is important*”). In the norms + value condition participants received the age-based norms feedback plus a value statement (“*Research has shown that knowing what others like you do and think in relation to a behaviour can be useful information in helping you decide for yourself”).*

#### Procedure

Participants were recruited to the online survey in March 2021. Past behaviour and demographic information (age, ethnicity, postcode) were assessed, and participants were randomized to condition and received information matched to their condition and age. Reactance and cervical cancer screening intention were then assessed.

#### Analyses

Chi square tests or ANOVA were used to compare the three conditions by participant age, recent screening, IMD quintile, and ethnicity to assess whether randomisation was successful. ANOVA was used to compare the three conditions on reactance.

The main analyses used a series of logistic regressions to predict the dichotomised intention to attend cervical screening based on condition, the effects of controlling for other variables, and any interactions between condition and other variables. The first analysis looked at differences between the control condition (coded 0) and the norms condition (coded 1). The second analysis looked at differences between the control condition (coded 0) and the norms + value condition (coded 1). The third analysis looked at differences between the norms condition (coded 0) and the norms + value condition (coded 1). In each regression, the condition variable was entered at step 1, other variables (age, recency of screening, IMD quintile, ethnicity) were entered at step 2, and the interactions between condition and other variables were entered at step 3. Model fits are reported plus for each variable unstandardized B values, standard errors (SE), significance (p), odds ratios (OR) and 95% confidence intervals (95%CI) around the odds ratios are reported.

### Results

#### Manipulation Check

There were no significant differences found between the three conditions for age, F(2, 296) = 1.56, *p* = .21; cervical screening recency, *χ*^2^ (2, *N* = 299) = 23.19, *p* = .20; white ethnicity, *χ*^2^ (2, *N* = 299) = 2.88, *p* = .24; or IMD quintile, *χ*^2^ (8, *N* = 299) = 7.56, *p* = .48. Therefore, study randomization to condition was successful.

There was also no difference in reactance by condition, *F*(2, 296) = 2.32, *p* = .10. This indicated that there was no impact of condition on reactance.

#### Logistic Regressions

The first logistic regression (upper panel, Table 1) indicated no significant difference between the norms and control conditions when considered alone (step 1, *p* = .61) or when also controlling for other variables (step 2, *p* = .68). In addition, there was no evidence of any interactions between condition and other variables (*ps* > .38).

**Table 1.** Logistic Regressions to Predict High (1) Versus Low (0) Intentions to Attend for Cervical Screening in Study 2.

	β	SE	*p*	OR	95%CI
Norms vs. Control conditions					
Step 1 (Δ χ^2^ (1) = 0.26, *p* = .612; Δ Nagelkerke *R*^2^ = .002)					
Condition	0.145	0.286	0.612	1.16	0.66, 2.02
Step 2 (Δ χ^2^ (4) = 6.25, *p* = .182; Δ Nagelkerke *R*^2^ = .041)					
Condition	0.120	0.293	0.683	1.13	0.64, 2.00
IMD quintile	0.227	0.127	0.074	1.16	0.98, 1.61
Age	−0.007	0.015	0.656	0.99	0.97, 1.02
Recency	0.630	0.404	0.119	1.88	0.85, 4.15
White ethnicity	−0.047	0.410	0.908	0.95	0.43, 2.13
Norms + Value vs. Control conditions					
Step 1 (Δ χ^2^ (1) = 6.18, *p* = .013; Δ Nagelkerke *R*^2^ = .041)					
Condition	0.711	0.288	0.014	2.04	1.16, 3.58
Step 2 (Δ χ^2^ (4) = 11.89, *p* = .018; Δ Nagelkerke *R*^2^ = .075)					
Condition	0.643	0.299	0.032	1.90	1.06, 3.42
IMD quintile	0.213	0.127	0.093	1.24	0.97, 1.59
Age	0.010	0.014	0.483	1.01	0.98, 1.04
Recency	1.129	0.398	0.005	3.09	1.42, 6.74
White ethnicity	0.112	0.441	0.800	1.12	0.47, 2.66
Norms + Value vs. Norms conditions					
Step 1 (Δ χ^2^ (1) = 3.95, *p* = .047; Δ Nagelkerke *R*^2^ = .026)					
Condition	0.566	0.286	0.048	1.76	1.01, 3.09
Step 2 (Δ χ^2^ (4) = 10.26, *p* = .036; Δ Nagelkerke *R*^2^ = .066)					
Condition	0.456	0.297	0.124	1.58	0.88, 2.82
IMD quintile	0.080	0.127	0.526	1.08	0.85, 1.39
Age	0.018	0.016	0.255	1.02	0.99, 1.05
Recency	1.150	0.407	0.005	3.16	1.42, 7.02
White ethnicity	0.101	0.466	0.829	1.11	0.44, 2.76

Note. For Norms versus Control Conditions: Step 3 Δ χ^2^ (4) = .79, *p* = .940; Δ Nagelkerke *R*^2^ = .005; For Norms + Value versus Control Conditions: Step 3 Δ χ^2^ (4) = 4.71, *p* = .319; Δ Nagelkerke *R*^2^ = .029; For Norms + Value versus Norms Conditions: Step 3 Δ χ^2^ (4) = 2.88, *p* = .579; Δ Nagelkerke *R*^2^ = .017.

The second logistic regression (middle panel, Table 1) indicated a significant difference between the norms + value and control conditions when considered alone (step 1, *p* = .01) and when also controlling for other variables (step 2, *p* = .03) with no evidence of any interactions between condition and other variables (*ps* > .17). Examination of the odds ratio (step 1, *OR* = 2.04) indicated that women were slightly more than twice as likely to be high intenders when in the norms + value condition compared to the control condition.

The third logistic regression (lower panel, Table 1) indicated a significant difference between the norms + value and norms conditions when considered alone (step 1, *p* = .05) that was rendered non-significant when also controlling for other variables (step 2, *p* = .12) with no evidence of any interactions between condition and other variables (*ps* > .27). Examination of the odds ratio (step 1, OR = 1.76) indicated that women were slightly less than twice as likely to be high intenders when in the norms + value condition compared to the norms condition.

### Discussion

Age-specific tailored norms information along with information highlighting the value of the normative information were effective in increasing the proportion of women with high screening intentions. This effect was significant compared to both the control and the norms conditions, although only in the former comparison did this difference remain significant when controlling for other variables. These differences did not significantly vary by age, ethnicity, IMD quintile or recency of cervical screening attendance. This study therefore demonstrates that a brief social norms intervention when supplemented by a value manipulation can significantly increase cervical screening intentions. We would note that our manipulation of value is similar to that used by Snyder and Kendzierski (1982) in relation to attitudes. In the Snyder and Kendzierski (1982) study it was shown that informing participants that their attitude was relevant to the decision increased the correspondence between attitudes and behaviour for those both low (i.e., look inwardly in making decisions) and high (i.e., look to their social environment in making decisions) in self-monitoring. This was interpreted as indicating that individuals are aware of their attitudes and can use this information in making decisions despite any dispositional preferences. In the current research we would suggest our findings support the idea that individuals can and do use normative information in making decisions particularly when the value of this information is highlighted.

## General Discussion

The two studies presented here investigated first whether individuals are poor at estimating the levels of cervical screening behaviour and attitudes in their peers, and second whether a manipulation of perceived norms could influence self-reported cervical cancer screening intentions. Study 1 supported social norms theory in that participants tended to underestimate the extent to which different groups of women participated in cervical screening compared to national statistics. These estimates were lower in younger women and those from more deprived areas but were not influenced by ethnicity or participants’ recency of screening. Study 2 demonstrated that exposure to a brief social norms intervention which informed participants about the national screening uptake in individuals from their age group, along with highlighting the value of norm-based messaging was effective in significantly increasing intentions to attend screening in future. Presenting age specific tailored norm information by itself was less effective in increasing intentions compared to control. Study 2 also indicated that the addition of some text to highlight the importance of the norm messaging (“Research has shown that knowing what others like you do and think in relation to a behaviour can be useful information in helping you decide for yourself”) was important for the intervention to be effective in influencing participant intentions to attend screening. Unsurprisingly, Study 2 demonstrated that participant intentions to attend screening were lower in individuals who were currently overdue for screening, or who had never attended screening before. However, the effect of the norm plus value condition remained when controlling for past screening behaviour, thereby demonstrating it as a potential brief, low-cost behaviour change intervention to encourage screening uptake in overdue/never screening women.

Individuals are influenced by behavioural norms (Berkowitz, 2005; Lewis & Neighbors, 2006; Schultz et al., 2007). Previous studies in this area have tended to focus on health risk behaviours and the present study supports the idea that this misperception of others behaviour also applies to cervical screening behaviour which may discourage performance of the behaviour (Perkins, 2014). Based on social norms theory, individuals are more influenced when norms are presented for others matching their own demographics.

A recent study demonstrated that general social norm messaging (“uptake is 8 out of 10“) increased bowel screening intentions in previously disinclined individuals (von Wagner et al., 2019). The manipulation study presented here (Study (2) support this study’s findings, but only when the value of the messaging was highlighted. Unlike the study by von Wagner et al., we did not question participant estimations of others’ behaviours in order to correct these. By highlighting these misperceptions, this might increase the potential for reactance effects, where individuals reject health messaging. We did not find different levels of reactance to the health messages compared with the control group who were presented with an unrelated message on workplace pensions.

In an attempt to avoid biased recruitment of individuals who were particularly interested in these specific behaviours, participants in both studies were not informed of the specific behaviours that would be questioned in the study. The demographic breakdown of participants also demonstrates that those recruited were from a range of socioeconomic and educational backgrounds.

### Strengths and Limitations

Both studies included hard-to-reach populations who typically do not engage in cervical cancer screening research (individuals from areas of the greatest deprivation and those who are overdue/had never attended screening before). We aimed to recruit a stratified sample in study 1 whereby 50% of the sample were from more deprived groups and 20% of the sample were from minority ethnic backgrounds, in order to match the general population of the UK.

However there were some sampling issues in study 2 including the fact that 86% of partcipants in Study 2 reported their ethnicity as White. Additionally, in study 2, while we were successful in recruiting 42% of the sample that were currently overdue/never screened, just under 68% of the participants reported high intentions to attend screening when next invited. This may have reflected the timing of the study (March 2021) where participants may have been overdue due to the COVID-19 pandemic, a reluctance to engage with health services during this time as well as dealing with the pandemic disrupting all aspects of daily life (O’Connor et al., 2020), rather than low intentions to attend cervical cancer screening.

### Conclusions

We demonstrate that women underestimate the proportion of other women attending cervical screening as well as that the majority of others have positive attitudes towards screening. A second study aiming to correct this misperception was found to be effective in increasing cervical screening intentions. This brief behaviour change intervention could be used to increase intentions to attend screening, including in individuals currently overdue for their cervical cancer screening. Such changes in intentions might be expected to be translated into greater attendance for cervical screening.

## Data Availability

Data available on reasonable request from the authors.
